# Chemokines Signature and T Cell Dynamics in Leishmaniasis: Molecular Insight and Therapeutic Application

**DOI:** 10.1017/erm.2024.36

**Published:** 2024-11-26

**Authors:** Shreya Upadhyay, Shashi Kumar, Vishal Kumar Singh, Rahul Tiwari, Awnish Kumar, Shyam Sundar, Rajiv Kumar

**Affiliations:** 1 Department of Medicine, Institute of Medical Sciences, Banaras Hindu University, Varanasi, India; 2 Centre of Experimental Medicine and Surgery, Institute of Medical Sciences, Banaras Hindu University, Varanasi, India

**Keywords:** chemokines, chemokine receptors, cutaneous leishmaniasis, desensitization, leishmaniasis, T-cell migration, visceral leishmaniasis

## Abstract

Leishmaniasis, caused by obligate intracellular *Leishmania* parasites, poses a significant global health burden. The control of *Leishmania* infection relies on an effective T cell-dependent immune response; however, various factors impede the host’s ability to mount a successful defence. Alterations in the chemokine profile, responsible for cell trafficking to the infection site, can disrupt optimal immune responses and influence the outcome of pathogenesis by facilitating parasite persistence. This review aims to emphasize the significance of the chemokine system in T cell responses and to summarize the current knowledge on the dysregulation of chemokines and their receptors associated with different subsets of T lymphocytes during Leishmaniasis. A comprehensive understanding of the dynamic nature of the chemokine system during Leishmaniasis is crucial for the development of successful immunotherapeutic approaches.

## Introduction

Leishmaniasis is a neglected tropical vector-borne disease caused by the protozoan parasite *Leishmania.* According to the World Health Organization (WHO), in 2022, Leishmaniasis was endemic in approximately 99 countries and territories out of 200 worldwide. It manifests in five different clinical forms, including visceral leishmaniasis (VL or kala-azar), post-kala-azar dermal leishmaniasis (PKDL), cutaneous leishmaniasis (CL), mucocutaneous leishmaniasis (MCL), and diffuse cutaneous leishmaniasis (DCL) (Ref. 1). Among these, VL is the most severe form, affecting approximately 90% of the global population and is primarily reported in seven countries: Brazil, India, South Sudan, Sudan, Ethiopia, Kenya, and Somalia (WHO report, 2018). VL affects the visceral organs of the host and is caused by the protozoan parasite *Leishmania donovani* in Asia, Africa, and the Middle East, and *Leishmania infantum* in South America and Europe. If left untreated, VL can be fatal. The disease is characterized by various symptoms, including splenomegaly (enlarged spleen), hepatomegaly (enlarged liver), pancytopenia (reduction in blood cell counts), hypergammaglobulinemia (elevated levels of gamma globulins in the blood), weight loss, weakness, and progressive anaemia (Ref. 2).

The chemokines and their receptors play a vital role in guiding immune cells to specific locations during homeostasis and inflammatory conditions. Chemokines, which are a type of cytokine, bind to their G-protein coupled receptors (GPCRs), known as chemokine receptors (CKRs), and initiate signalling through coupled heterotrimeric G-proteins (Ref. 3). This signalling pathway leads to the activation of integrins, enabling leukocytes to firmly adhere to endothelial cells and extravasate into the tissue microenvironment (Ref. 4). Chemokine receptors are designated based on the type of chemokine(s) they bind, such as CXC, CC, XC, and CX3C, followed by ‘R’ (for a receptor) and a number indicating the order of discovery. The chemokine system plays a crucial role in immune cell migration and the composition of immune cells at a specific site depends on various factors, including chemokine expression. This composition of immune cells also influences the host’s susceptibility to infection. During inflammation, various types of immune cells, including neutrophils, macrophages, and lymphocytes, as well as non-immune cells such as endothelial cells, epithelial cells, fibroblasts, and adipocytes, produce chemokines. This results in the migration of different cell types, such as macrophages, neutrophils, and T cells, to the specific location of inflammation (Refs. 5, 6). The secretion of cytokines from these cells in the inflamed zone affects the behaviour of infiltrating cells and disease progression (Ref. 7). For instance, CXCL8 is secreted by endothelial cells, and wounded epithelial cells recruit neutrophils which can further release some more CXCL8 and attract even more neutrophils, and other types of leukocytes to the inflamed zone (Refs. 6, 8, 9). T lymphocytes, a subset of immune cells, have a central role in combating intracellular infections and coordinating adaptive immune responses. T lymphocytes can produce proinflammatory or anti-inflammatory cytokines and can eliminate unwanted cells (Ref. 10). They express a range of chemokine receptors on their surface and also produce various chemokines, including CXCR3, CCR5, CCR4, CCR8, CCL3, CCL4, CCL5, CXCL8, etc. (Table 1).

**Table 1.** CD4^+^ T cell subsets expressing chemokine receptors and their subsequent ligands

No.	CKRs types	Chemokine receptors (CKRs)	Chemokines (corresponding ligands)	T-subsets expressing CKRs	Inflammatory conditions	References
**1**	CCRs	CCR1	CCL3, CCL5–9, CCL13–16, CCL23	Th1, Th2, Th9, Th17, Trm	rheumatoid arthritis, allergic rhinitis, tumour	(11; 12)
2		CCR2	CCL2, CCL7, CCL8, CCL12, CCL13	Th1, Treg, Th17	tumour, melanoma, pancreatic cancer	(13; 14; 15; 16; 17; 18; 19; 20; 21)
3		CCR3	CCL5–8, CCL11, CCL13, CCL15, CCL24, CCL26	Th2, Th9, Treg	atopic dermatitis, cancer, experimental colitis, allergic inflammation	(13; 14; 15; 16; 17; 18; 19; 22; 23)
4		CCR4	CCL17, CCL22	Th2, Treg, Th17, Th22, CD8	melanoma, atopic dermatitis, cancer, allergic inflammation	(13; 14; 15; 16; 17; 18; 19; 20; 21; 24)
5		CCR5	CCL3–5, CCL11, CCL14, CCL16	Th1, Th9, Treg, Th17	melanoma, atopic dermatitis, HIV-infection	(21; 24; 25)
6		CCR6	CCL20	Th17, Treg, Th9, Tfh, Th22	melanoma, tumour, pancreatic cancer, lymph-borne pathogenic response, skin inflammation	(13; 14; 15; 16; 17; 18; 19; 20; 21; 26; 27; 28; 29)
7		CCR7	CCL19, CCL21	Tcm, Trcm, Treg, Naïve T cell	melanoma, homeostasis, self-tolerance	(21; 30)
8		CCR8	CCL1, CCL18	Th2, Treg, Skin CD4 Trm	allergic inflammation, lung cancer, skin disease	(24; 31; 32)
9		CCR9	CCL25	Th17, Th22	viral infection, intestinal inflammation	(33; 34)
10		CCR10	CCL27	Th17, Th22	malignant ascites, skin pathophysiology	(13; 14; 15; 16; 17; 18; 19; 20; 35; 36; 37; 38)
11	CXCRs	CXCR1	CXCL8, CXCL6, CXCL1	CD4, CD8	leukaemia, homeostasis, viral and tumour inflammation, allergic disease	(39; 40; 41; 42; 43; 44; 45; 46)
12		CXCR2	CXCL1–3, CXCL5–8	CD4, CD8	multiple sclerosis, cancer, experimental autoimmune encephalomyelitis (EAE)	(39; 42; 43; 47; 48)
13		CXCR3	CXCL9–11	Th1, Treg, Th9, Tfh, Th17, CD8 Tcm & Tem	melanoma, atopic dermatitis	(13; 14; 15; 16; 17; 18; 19; 20; 21; 43; 49)
14		CXCR4	CXCL12	CD4, CD8	homeostasis, HIV infection, tumour, prostate cancer, pancreatic cancer	(13; 14; 15; 16; 17; 18; 19; 20)
15		CXCR5	CXCL13	Th17, Tcm, Tem, Tfh, CD8	humoral responses, rheumatoid arthritis, autoimmune disease	(50; 51; 52)
16		CXCR6	CXCL16	Th1, Th17, CD8	inflamed human liver, experimental autoimmune encephalomyelitis (EAE), Alzheimer’s disease,	(21; 29; 53; 54)

[CCL = chemokine ligand; CXCL = C-X-C motif chemokine ligand; CCR = β-chemokine receptors; CXCR = α-chemokine receptors; Tcm = central memory T cells; Tem = effector memory T cells; Tfh = follicular helper T cells; Treg = regulatory T cells; Th = helper T cells]

However, the chemokine system associated with T cells, particularly in Leishmaniasis, has received limited attention. Understanding the complex interactions between the chemokine system and T cells is crucial to elucidate the impaired migration and functioning of immune cells during *Leishmania* infection. This understanding can contribute to the identification of potential drug targets against chemokines and chemokine receptors, facilitating the development of novel therapeutic strategies.

## T-cell associated chemokine system: at the crossroads of infection or protection

The chemokine profile plays a critical role in the migration of immune cells during homeostatic and inflammatory conditions (Ref. 55). Chemokine receptors (CKRs) are expressed on the surface of immune cells and exhibit differential expression patterns (Ref. 56). The promiscuous nature of the chemokine system allows multiple chemokines to bind to a single receptor, and conversely, a single chemokine can interact with multiple receptors (Ref. 57). This complex interaction between chemokines and receptors influences the migratory behaviour and functional consequences of immune cells (Refs. 58, 59). Chemokines belonging to the CC family, such as RANTES (CCL5), can bind to multiple chemokine receptors, including CCR1, CCR3, and CCR5. Similarly, CC chemokine receptor 5 (CCR5) can interact with different chemokines like MIP-1β, MIP-1α, and RANTES (Ref. 60). This promiscuity allows for versatile chemokine-receptor interactions, expanding the repertoire of migratory signals that immune cells can respond to. The expression pattern of chemokine receptors on the cell surface determines the migratory behaviour of immune cells in response to specific chemoattractant sources. Instead of directly migrating to a specific site, cells pass through different zones expressing different chemokines. This multistep directional migration is guided by the combinatorial expression of chemokine receptors on the cell surface (Ref. 61). For example, naïve T cells require CCR7 to migrate to the T cell zone, expressing CCL19, and once there, desensitization or downregulation of CCR7 allows them to migrate to the B-cell zone, guided by CXCR5/CXCL13 axis (Ref. 62).

The expression of chemokine receptors is tightly regulated during cell development and differentiation (Ref. 63). This regulation allows for the distinction of different forms of CD4^+^ and CD8^+^ T cells, such as naïve T cells, effector T cells, and memory T cells, based on the specific chemokine receptors they express. Each T cell subset uniquely expresses various chemokine receptors that define its identity and functional characteristics (Ref. 10). The host employs various strategies to combat pathogenesis during infection. The development of resistance in the host largely depends on the orchestrated response of cells that possess the ability to eliminate pathogens. Chemokines play a crucial role in directing selective cell migration towards the site of infection. Depending on the specific chemokine signals present, the host may mount a protective response or experience tissue damage (Ref. 64). The chemokine receptors associated with different subsets of T lymphocytes under various inflammatory conditions, have been summarized in Table 1.

## Migratory control over naïve and central memory T cells

Naïve T cells (Th0) and central memory T cells (Tcm) express crucial homing receptors, such as CCR7 and CXCR4, which are involved in their migration to secondary lymphoid organs (SLOs) where they can actively participate in immune surveillance and responses (Refs. 65–67). Naïve T cells are those that have not been previously exposed to antigens, circulate in the bloodstream and travel to lymph nodes, where they scan for antigens presented by antigen-presenting cells (APCs) to initiate an immune response. CCR7 facilitate rolling over the endothelium of blood vessels during transmigration. Homeostatic chemokines CCL19 and CCL21, which are secreted by high endothelial venules (HEV), stimulate the CCR7 receptor on T cells (Ref. 68). The interaction between CCR7 and its ligands increases the affinity of the integrin LFA-1 (found on lymphocytes and other leukocytes) for its ligand ICAM-1 (expressed on HEV). This firm attachment to the endothelium enables T cells to migrate through the HEV and enter the lymph node (Refs. 69, 70). Experimental studies using mutant mice lacking CCR7 (CCR7^−/−^) have demonstrated impaired immunogenic responses due to restricted entry of lymphocytes from the bloodstream to SLOs (Ref. 71). Similarly, Tcm cells also express CCR7 which facilitates its retention in SLO. Another homing receptor, CXCR4 interact with CXCL12 (SDF-1) and is involved in memory T cell maintenance, cell growth, cell survival, and the recirculation of T cells within SLOs. Bone marrow stromal cells express CXCL12 which attracts T cells expressing CXCR4 on its surface (Refs. 72, 73). Its expression is reduced once T cells are activated (Ref. 74). CXCR4 is a remarkable marker expressed constitutively on both naïve CD4^+^ and CD8^+^ T cells, but predominantly on naïve and central memory CD8^+^ T cells (Refs. 72, 75, 76).

CCR7 is highly expressed on resting naïve CD4^+^ T cells (CD45RA^+^ CCR7^+^), however, most activated T cells lack CCR7 on their surface, and if they do, it is expressed at a very low level (Ref. 77). Tcm cells do not possess effector functions but can differentiate into effector memory T (Tem) cells upon antigenic stimulation having lower CCR7 but upregulated some other chemokine receptors like CCR5, CXCR3, and CCR4 (Refs. 78, 79). This transition allows them to migrate to peripheral tissues to provide robust immune responses rather than to rest within the lymphoid tissues.

Similarly, CD8^+^ T cells also express CCR7 on their surface and migrate towards SLOs, like CD4^+^ T cells as discussed earlier (Ref. 80). CXCR4 is a remarkable marker expressed constitutively on both naïve CD4^+^ and CD8^+^ T cells, but predominantly on CD8^+^ T cells (Ref. 75). It interacts with its ligand, stromal cell-derived factor 1 (SDF-1 or CXCL12), and regulates the migration of CXCR4^+^ T cells by facilitating their adhesion to the venules of SLOs. The presence of CXCR4 has been discovered to provide essential signals for the survival of thymocytes during their maturation process. Disrupting the function of CXCR4 has an impact on thymic development (Ref. 81). CXCL12/CXCR4 signalling is crucial for TCR-induced immunological synapse development, early signalling molecule phosphorylation, and thymic β selection (Ref. 82). CXCR4 mediates the migration of naïve and central memory (Tcm) CD8^+^ T cells to the bone marrow and is critical for the homeostatic proliferation of CD8^+^ Tcm cells. It also maintains the reservoir of memory CD8^+^ T cells (Ref. 72). Their expression decreases during differentiation into effector memory cells (CD8^+^ Tem) as negatively correlated with perforin expression (Ref. 75).

## Migratory control over effector memory T cells

As naïve T cells differentiate into effector T cells, they begin to express additional chemokine receptors (Table 1) that are necessary for their migration and positioning within target tissues (Refs. 83, 84). Effector memory T cells (Tem) are CCR7^low^ and express other chemokine receptors that facilitate their circulation in the peripheral blood and migration to inflamed tissues, where they can exert their protective functions against infections (Ref. 85).

Different subsets of CD4^+^ effector cells, such as Th1 and Th2 cells, express distinct arrays of chemokine receptors. Th1 cells preferentially express CCR5 and CXCR3, while Th2 cells, on the other hand, preferentially express CCR3, CCR4 and CCR8 (Refs. 86, 87) which are involved in their migration to inflamed tissues. CXCR5 is a chemokine receptor that directs the migration of T cells into B cell follicles. While subsets of both CD4^+^ and CD8^+^ T cells express CXCR5, its high expression is found on T follicular helper cells (Tfh), a subset of CD4^+^ T cells (Ref. 88). The ligand for CXCR5, CXCL13 is released from ‘B cell zones’ in secondary lymphoid organs and guides the migration of Tfh cells towards B cell follicles, where they assist in affinity maturation (Ref. 89). Deletion of CXCR5 or CXCL13 in mice leads to altered and impaired microarchitecture of secondary lymphoid organs (Refs. 90, 91). CXCR5^+^ central memory T cells (Tcm) play a crucial role in the generation of antibody-mediated secondary immune responses (Ref. 92). The immunosuppressive CD25^+^ regulatory T cells (Tregs) were found to be associated with many C-C chemokine receptors such as CCR4, CCR5, CCR6, CCR7 & CCR8 but majorly express CCR4 and CCR8 (Refs. 93–95). Previously, it was found that CXCR4 expression decreases with T cell activation, however, subsequent discoveries have also shown that its expression increases on CD4^+^ T cells in diseased conditions as reported in HIV-infected patients where it acts as a coreceptor for HIV-entry (Refs. 74, 76, 96).

Effector CD8^+^ T cells express chemokine receptors such as CXCR3, CXCR6, CCR4, CCR6, CCR9 and CCR10, which direct their migration to specific tissues during inflammatory responses (Refs. 80, 97). IFN-γ producing CD4^+^ T cells affect the recruitment of effector CD8^+^ T cells by upregulating the production of CXCL9 and CXCL10 (ligands for CXCR3) at the site of infection (Ref. 80). CXCR3^high^ has been found to be a determination factor of cytotoxic response, as studied during influenza pathogenesis (Ref. 58). CXCR3 expression is induced on naive CD8^+^ T cells upon activation and remains preferentially upregulated on effector CD8^+^ T cells. CXCR3 is involved in the migration of CD8^+^ T cells to inflammatory sites. Antigen-specific CD8^+^ T cells that lack CXCR3 skewed towards more memory cells with decreased activation properties and fewer short-lived effector cells (Refs. 97, 98). CCR9 promotes migration to the gut, while CCR10 facilitates migration to the skin (Ref. 99), indicating that the draining lymph node plays a significant role in determining the migratory properties of activated CD8^+^ T cells, guiding them toward specific locations.

In summary, the expression of specific chemokine receptors on effector memory T cells determines their migratory behaviour and allows them to migrate to the appropriate tissues during an immune response. The differential expression of chemokine receptors on different subsets of T cells contributes to their specialized functions and distribution within the body.

## Chemokine signalling

**Figure 1.**
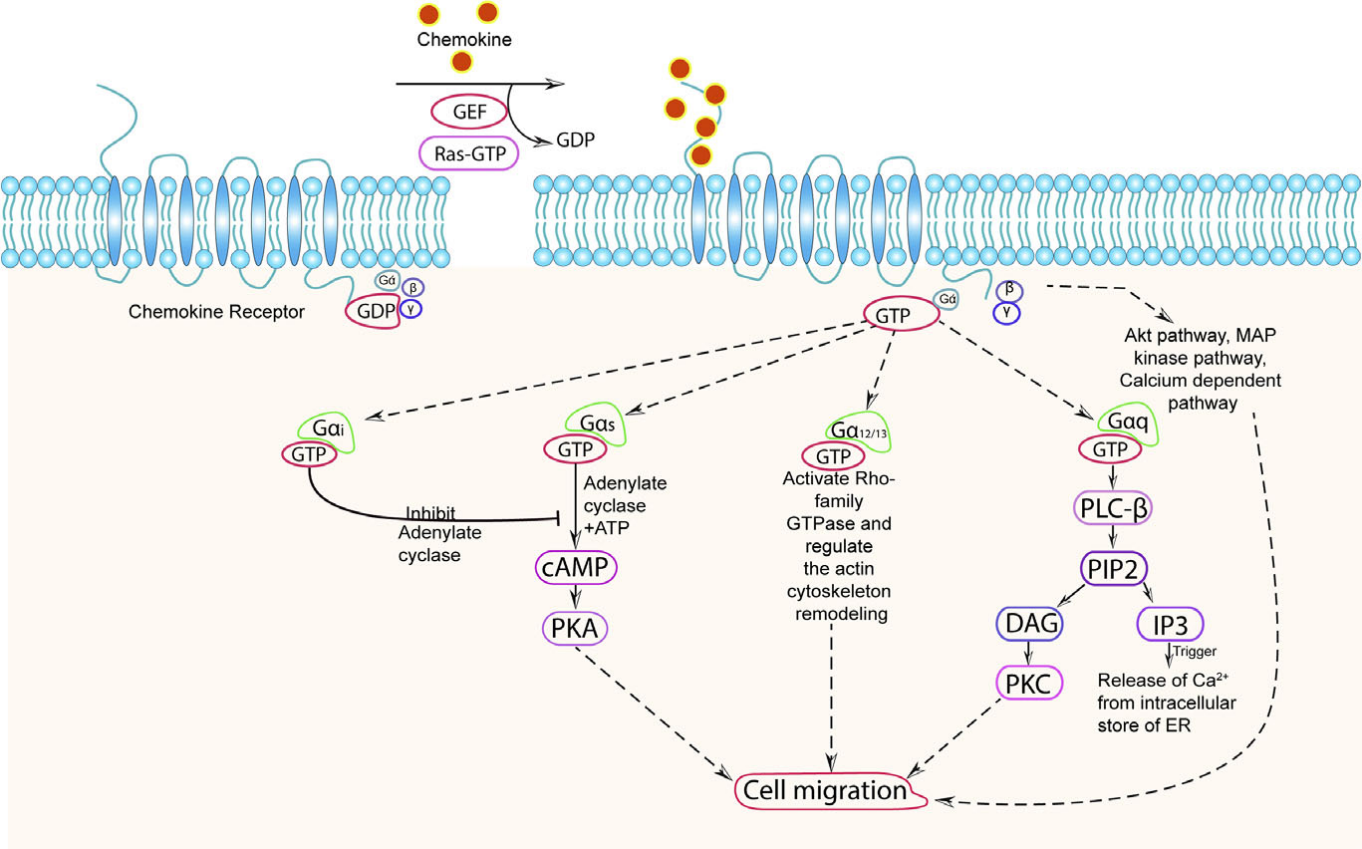
Chemokine signalling pathway. Chemokine receptor (CKR) remains in an inactive stage in which chemokine is not associated with it, and G-protein is in an inactive state and bound with GDP. CKR on interaction with specific chemokine triggers the activation of the bound heterotrimeric G-protein composed of αβγ subunits which leads to an exchange of GDP with GTP and dissociation of the heterotrimeric G protein complex into Gα and Gβγ subunits where GTP remains attached to Gα subunit. Depending on the nature of the inducing signal and types of Gα protein, different signalling pathways get activated. (a) Gα_i_ inhibits the activity of adenylate cyclase enzyme and reduces the cAMP generation; (b) Gα_s_ stimulate the activity of adenylate cyclase enzyme and stimulates the production of cAMP which further activates PKA; (c) Gα_12/13_ activates rho-family GTPase and regulate the actin cytoskeleton remodelling; (d) Gα_q_ (or Gβγ) activate PLC-β enzyme which cleaves PIP2, located in the plasma membrane, into DAG molecules and intracellular secondary messenger IP3. DAG further activate PKC and IP3 binds to its receptor on endoplasmic reticulum (ER) causing Ca^2+^ release into the cytoplasm; (d) Gβγ can also activate the Akt pathway, MAP kinase pathway, and Ca^2+^ dependent pathway. (d) Both the Gα and Gβγ subunits are capable of initiating a downstream signalling cascade that results in a range of cellular activities, including changes in cytoskeleton dynamics and cell migration that ultimately regulate the physiological and pathological response of the cells.

Chemokine receptors (CKRs) are a type of G protein-coupled receptors (GPCRs) that play a crucial role in cell signalling. The signalling of CKRs involves various molecules, including heterotrimeric G proteins, G protein receptor kinases (GRKs), and β-arrestins. These components work together to initiate and regulate signal transduction pathways, leading to a wide range of biological functions (Ref. 100). When a specific stimulus binds to a heptahelical chemokine receptor, it activates specific heterotrimeric G proteins. These G proteins consist of an alpha subunit (Gα) and a beta-gamma subunit (Gβγ). Different Gα subunits have been identified on the basis of sequence and functional similarities (Table 2) – stimulatory subunit (Gα_s_), inhibitory subunit (Gα_i_), Gα_12/13_, and Gα_q_ (Ref. 107). Initially, the Gα subunit is bound to GDP (guanosine diphosphate), but upon stimulation, guanine nucleotide exchange factors (GEFs) stimulate the exchange of GDP for GTP (guanosine triphosphate) on the Gα subunit. The binding of GTP to Gα leads to its activation and activated Gα subunits can then interact with various downstream effectors like adenylate cyclase (AC), GTPase of rho-family, protein kinase A (PKA), protein kinase C (PKC) and so forth in order to perform effector functions, including cell migration (Refs. 101, 102, 108–110). For example, Gαq can activate an enzyme called phospholipase C (PLC), which is associated with the cell membrane. PLC cleaves phosphatidylinositol (4,5)-bisphosphate (PIP2) into two-second messenger molecules: diacylglycerol (DAG) and inositol triphosphate (IP3). DAG activates protein kinase C (PKC), while IP3 triggers the release of calcium ions from intracellular stores, such as the endoplasmic reticulum (Refs. 105, 111, 112). These events initiate multiple signalling cascades that ultimately lead to various cellular responses, including actin polarization and chemotaxis (Figure 1) (Refs. 107, 113).

**Table 2:** G alpha protein subunits and their corresponding signalling pathways

S.No.	G alpha subunit	Signalling pathway
**1.**	Gα_s_ (‘s’ stimulatory)	Activate adenylate cyclase and cAMP-dependent protein kinase A (PKA) (101; 102)
**2.**	Gα_i_ (‘i’ inhibitory)	Inhibit adenylate cyclase and protein kinase A (PKA) (101; 103;104)
**3.**	Gα_q/11_	Stimulate phospholipase C (PLC-β) to cleave PIP_2_ into DAG and IP_3_ and activate Protein Kinase C (PKC) and Ca^2+^ dependent pathway (105)
**4.**	Gα_12/13_	Activate Rho-family GTPase and regulate the actin cytoskeletal remodeling (106)

To regulate the ongoing signalling, there is a regulator of G protein signalling (RGS) proteins that act as GTPase-activating proteins (GAPs) for Gα subunits. They facilitate the hydrolysis of GTP bound to Gα, thereby switching off the ongoing signalling processes. On the other hand, the Gβγ dimer, which remains bound together, acts as a signalling molecule itself. It can initiate signalling pathways independently and also regulate the activity of Gα subunits. Some of the pathways regulated by Gβγ include the Akt pathway, MAP kinase pathway, and calcium-dependent pathway, which can lead to cellular responses like cell migration (Ref. 114). Gβγ subunit mainly negatively regulates Gα subunit when bound with it. Intracellular GPCR kinases (GRKs) play a role in the regulation of CKRs. Upon continuous stimulation with chemokines, GRKs phosphorylate the CKRs. This phosphorylation allows for the binding of arrestin proteins, leading to the desensitization or internalization of the CKRs. This process can ultimately result in the degradation of the receptors or their recycling back to the cell surface. Different chemokines can activate the same CKR through different GRKs. For example, CCR7 can be activated by both GRK3 and GRK6 in response to CCL19, while CCL21-induced CCR7 signalling is mediated only by GRK6 (Ref. 115). A phenomenon known as oligomerization, the formation of complexes between either the same or different CKRs, has also been reported. This can lead to altered receptor activity and crosstalk between signalling pathways, which may affect normal signalling and result in a variety of cellular responses, including the regulation of cell migration (Ref. 116). As studied in the case of CCR7, oligomerization is necessary for effective cell migration. If oligomerization were to somehow fail, cell movement would be hampered (Ref. 117).

## Role of T cells during Leishmaniasis

**Figure 2.**
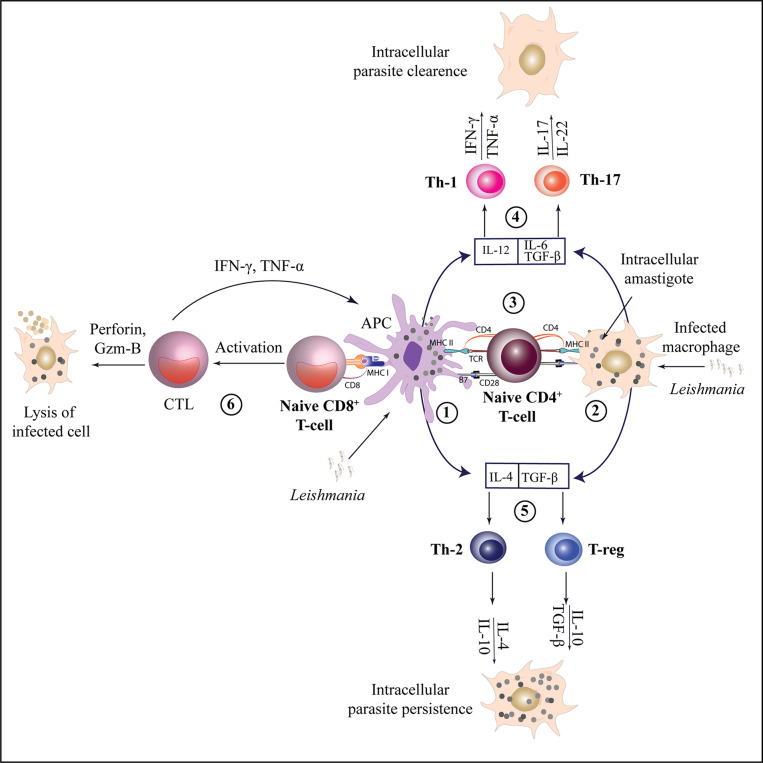
Activation and differentiation of CD4^ + ^T and CD8^ + ^T cell subsets during leishmaniasis. *Leishmania* antigens are presented by APCs or infected macrophages (1,2) to naïve CD4^+^ T cells through MHC class II molecules, leading to their activation. Depending on the cytokine environment, naïve CD4^+^ T cells can differentiate into various T-helper subsets (3). Interleukin-12 (IL-12) facilitates the differentiation of Th1 cells, which produce IFN-γ and TNF-α, promoting the clearance of intracellular parasites. Th17 cells, on the other hand, produce IL-17 and IL-22, contributing to anti-leishmanial and inflammatory responses (4). Th2 cell differentiation occurs under the influence of IL-4, leading to the production of IL-10 and IL-4 which can result in parasite persistence by inhibiting macrophage activation. Similarly, TGF-β promotes the differentiation of T-regs, which produce IL-10 and TGF-β, contributing to immune regulation and further supporting parasite persistence (5). Naïve CD8^+^ T cells are activated via MHC class I molecules and can differentiate into CTLs, producing perforin and granzyme B to target infected cells. They also produce IFN-γ and TNF-α, which support the Th1 response for effective parasite clearance (6). [CTL- Cytotoxic T lymphocytes, APC- Antigen presenting cell, MHC- Major Histocompatibility complex, Gzm B-Granzyme-B, TGF β- transforming growth factor-β, T-regs - Regulatory T cells].

The orchestration of T lymphocytes on the targeted site plays a central role during adaptive immunity. An optimal T cell-dependent immunoprotective response is essential to combat infection caused by obligate intracellular *Leishmania* parasites in the mammalian host. Different subsets of T cells have been discovered to play various roles in different clinical forms of Leishmaniasis, highlighting the importance of understanding the types of T cells that exhibit protective and destructive responses during infection (Figure 2).

## CD4^+^ T cells

CD4^+^ T cells, a major group of T cells, provide protection to the host during leishmaniasis which relies on the expression of various antiparasitic molecules (e.g. reactive oxygen species, nitric oxide) in phagocytic cells that get activated on IFN-γ productions (Ref. 118). Various subsets of CD4^+^ T cells, including Th1, Th2, Th17, Th22, Th9, Treg and Tfh cells, have been identified based on their distinct cytokine profiles (Table 3). These subsets are responsible for different immune responses and can determine resistance or susceptibility to *Leishmania* infection, depending on which subset dominates the infected site. Treg cells possess immunosuppressive properties during infection. They play a regulatory role in dampening immune responses and can contribute to the persistence of the parasite (Ref. 129).

**Table 3.** Cytokine profiles of different CD4^+^ T cell subsets during Leishmaniasis

S.No.	**CD4**^**+**^ **T cell subsets**	Cytokine profiles in *Leishmania-infected* patients	References
**1**	Th1	IFN-γ, TNF-α, IL–12	(119)
**2**	Th2	IL–4, IL–5, IL–13, IL–10	(120)
**3**	Tfh	IL–21, IL–4	(121; 122; 123)
**4**	Th17	IL–17, IL–22, IL–9	(119; 124; 125)
**5**	Th22	IL–22	(126)
**6**	Th9	IL–9	(127)
**7**	Treg	IL–10, TGF-β, IL–35, IL–9	(128; 120)

In VL, Th1 cells produce pro-inflammatory cytokines such as IFN-γ and TNF-α, which induce phagocytic activity and control parasitic growth while Th2 cells produce a higher level of IL-4, IL-5, IL-13, and IL-10 that leads to susceptibility towards infection (Refs. 130, 131). Another proinflammatory subset of CD4^+^ T cells, Th17 produces IL-17 and IL-22 that recruit neutrophils and inflammatory cells at the inflammatory site, thus playing a protective role during VL (Refs. 132, 133). It was observed that the cytokines IL-10, TGF-β and IL-35 released by these cells hinder the functioning of IFN-γ, TNF-α and IL-17 during chronic VL as studied on *Leishmania donovani* infected mice model (Refs. 134, 135). T follicular helper (Tfh) cells, an important CD4^+^ T cell subset that regulates B lymphocyte activation during humoral immune responses, produce IL-21 and IL-4 (Ref. 121). It has been found that IL-21 mRNA expression was upregulated in CD3^+^ T cells of VL patients which is responsible for the expansion of IL-10-producing cells (Refs. 136, 137). As, IL-21 also assists in antibody production, their increased level in the serum of chronic VL patients may be responsible for generating autoantibodies (Refs. 138, 139). Th9 subset secretes IL-9 during infection. CD4^+^ T cells releasing IL-9 have been found to be upregulated in human VL during the acute phase and lead to immunopathogenesis (Ref. 140).

In CL caused by *Leishmania (V.) braziliensis*, patients with active lesions exhibit a mixed Th1/Th2 response, producing cytokines like TNF-α, IFN-γ, IL-12, IL-4, and IL-1. However, individuals who have been cured of the infection primarily produce IFN-γ (Th1 response), which is associated with a protective immune response (Ref. 141). Although IFN-γ and TNF-α provide protection to the host against leishmaniasis their overproduction may cause tissue damage (Ref. 142). IL-22, released by Th22 and Th17 cells, found to provide protection against tissue destruction during CL (Ref. 143). IL-17 was considered a predictive marker of disease progression in *L. guyanensis-infected* CL patients (Ref. 144). High production of IL-17 cytokine has been directly associated with disease severity in CL (Ref. 145). Primarily IL-9 is produced by the Th9 subset, but Th17 and Treg cells also produce this cytokine at a low level and are involved in CL pathogenesis (Refs. 120, 128).

During PKDL, there is an increase in the production of Th1-cell-specific cytokines, namely IFN-γ, TNF-α and IL-12, as well as IL-17A, IL17F and IL22 specific to Th17 cells show a protective role during infection. IL-17 may contribute to resistance by increasing the production of TNF-α, NO, and antimicrobial peptides (like β-defensin) in conjunction with IL-22 (Ref. 131). Th2 cells produce a higher level of IL-4, IL-5, IL-13, and IL-10 that leads to susceptibility towards infection and promote parasite persistence during PKDL. The progression of VL to PKDL is associated with the overproduction of Th2-related cytokines in the skin (Ref. 120). The simultaneous overproduction of IL-10 diminishes the effectiveness of IFN-γ and TNF-α (Ref. 146). It was also found that the patients with PKDL had lower levels of serum IFN-γ, IL-10, and IL-6 compared to VL patients and comparable levels to healthy persons. However, the levels of TNF-α in PKDL patients were considerably higher than in VL patients or healthy participants (Ref. 147). Different kinds of PKDL have varying levels of these cytokines, polymorphic PKDL had greater serum levels of IFN-γ and IL-10 than macular PKDL, while macular lesions had lower levels of IFN-γ and TNF-α than nodular PKDL (Ref. 131).

## CD8^+^ T cells

The role of CD8^+^ T cells in leishmaniasis has received relatively less attention compared to CD4^+^ T cells. Nonetheless, studies have demonstrated that CD8^+^ T cells, specifically the Tc1 subset, do play a protective role in protozoan infections, including leishmaniasis. CD8^+^ T cells exert their protective effects through various mechanisms. They produce inflammatory molecules such as IFN-γ and TNF-α, which contribute to the activation of macrophages and the control of intracellular pathogens like *Leishmania.*

In VL, CD8^+^ T cells play a role in defending against the development of the disease. They secrete IFN-γ, perforin, and granzyme, which contribute to the control of *Leishmania* infection (Refs. 148, 149). However, during the progression of human VL, there is often a depletion of CD8^+^ T cells possessing anergic phenotype, which reduces their protective potential against the parasite (Ref. 150). There are two distinct groups of CD8^+^ T cells have been identified, one is CD8^low^ which was present during onset and VL progression, and the other one is CD8^high^ which increases after the cure of the disease (Ref. 151). Despite the challenges observed in human VL, studies in mouse models have shown promising results regarding CD8^+^ T cell-based vaccines. These vaccines rely on the chemokine CXCL10, which plays a crucial role in attracting CD8^+^ T cells to the sites of infection. By enhancing the recruitment and activation of CD8^+^ T cells, CXCL10-based vaccines have demonstrated effectiveness in reducing the parasitic burden in organs (Ref. 152).

The production of IFN-γ, TNF-α and cytolytic molecules by CD8^+^ T cells play a protective role during CL also (Refs. 153). The cytolytic genes are highly expressed in lesions and are positively correlated with the recruitment of granzyme B^+^ CD8^+^ T cells (Ref. 154). CD8^+^ T cells contribute to resistance against *L. major* infection by increasing the development of Th1 cells and suppressing the development of Th2 cells, via the production of IFN-γ (Ref. 155). Additionally, CD8^+^ T cells are also responsible for the host immunopathology during CL (Ref.^156^). A previous report found an association between granzyme B and disease outcome. It was observed that inhibiting the granzyme release from CD8^+^T cells during CL reduces disease severity (Ref. 157). CD8^+^ T cell-mediated pathology has been linked with the induction of inflammasome NLRP3 formation and release of IL-1β which is confirmed by the increased level of this cytokine in the lesions of patients infected with *L. braziliensis* (Ref. 158). This suggests that CD8^+^ T cells possess protective as well as immunopathogenic nature during *Leishmania* infection.

The frequency of IL-10-producing CD8^+^ T cells was considerably elevated in individuals with PKDL caused by *L. donovani*, but it decreased after successful treatment (Ref. 159). Increased expression of exhaustion markers such as programmed death-1 (PD-1), while reduced expression of perforin and granzyme was also observed at lesional site (Ref. 160). This implies that the conditions are favourable for the survival of parasites and lead to the progression of diseases.

## γδ T cells

Gamma delta T cells (γδ T cells) account for 2–5% of the overall cell population in healthy persons and possess a γδ T-cell receptor (TCR) on their cell surface rather than αβ TCR chains as found in the case of CD4^+^ and CD8^+^ T cells. A previous study demonstrated that mice infected with *L. major* subcutaneously exhibited elevated levels of γδ T cells in the spleen and draining lymph nodes of both susceptible BALB/c and resistant CBA/J mice. This suggests that γδ T cells are involved in protective inflammatory responses associated with the infection by promoting granuloma formation (Refs. 161–163). In VL patients, elevated γδ T cells were observed to stimulate the proliferation and differentiation of B cells which is achieved through the secretion of growth factor (BCGF) and differentiation factor (BCDF). This results in abnormalities in humoral immune responses and hypergammaglobulinemia, suggesting an immuno-suppressive and pathogenic response (Refs. 164). In another study of VL patients infected with *L. donovani*, a substantial production of IL-10 was found which suggests an immunomodulatory function of γδ T cells (Refs. 165). In an experimental model of C57BL/6 mice infected with *L. donovani*, it was shown that IL-17, which is generated by γδ T cells, has an inhibitory effect and restricts the proliferation of parasites in the liver (Ref. 166).

## Natural killer T cells (NKT)

NKT cells are specialized lymphocytes that share surface markers and functional characteristics with both natural killer cells (NK) and T cells (Ref. 167). They may express CD4 or CD8 markers on their surface and secrete IFN-γ, TNF-α, IL-4, IL-10, and IL-13 and constitute 0.1–0.5% of peripheral blood leukocytes (Refs. 168, 169). IFN-γ-producing CD8^+^ NKT cells were shown to be protective in nature, whereas CD4^+^ NKT cells expressing CD25, Foxp3 and IL-10 were found to be pathogenic during *L. donovani* infection (Ref. 170). These CD4^+^ NKT cells accumulate at the infection site and it may be due to the expression of CCR5 on its surface during the infection (Ref. 171). In a previous study on peripheral blood of VL patients, it was observed that CD8^dim^ CD56^+^ NKT cells are the subset which express more granzyme B and are more cytotoxic than CD8^bright^ CD56^+^ NKT cells (Ref. 172).

In CL, CD3^+^ CD56^+^ CD8^+^ NKT cells were also found to be protective in nature and shown to be associated with a cytotoxic response against *L. braziliensis* (Refs. 171, 173). In CD1d^−/−^ and Jα18^−/−^ mice, which lack NKT cells, exhibited a delay in clearing >10^^6^ *L. major* parasites during infections (Ref. 174).

However, despite the presence of the defensive properties of CD4^+^ T cells and CD8^+^ T cells, immune responses are ineffective in controlling parasitic growth and thus disease progression occurs during chronic infections. Furthermore, hyporesponsive T cells expressing several exhaustion markers (eg. PD-1, CTLA-4, LAG-3, TIM-3) lead to ineffective immune responses and high parasitic load that depends on infection duration and host immunity (Ref. 175). By understanding the role of chemokines and their receptors associated with different T cell subsets during leishmaniasis, we can get valuable information on the key factors driving disease progression and prognosis, potentially leading to better clinical management of the disease. Targeting specific chemokines and their receptors holds the potential for modulating T-cell responses and enhancing protective immunity against *Leishmani*a infection.

## Hepatic granuloma formation during VL is a function of T-cell-associated chemokine profile

**Figure 3.**
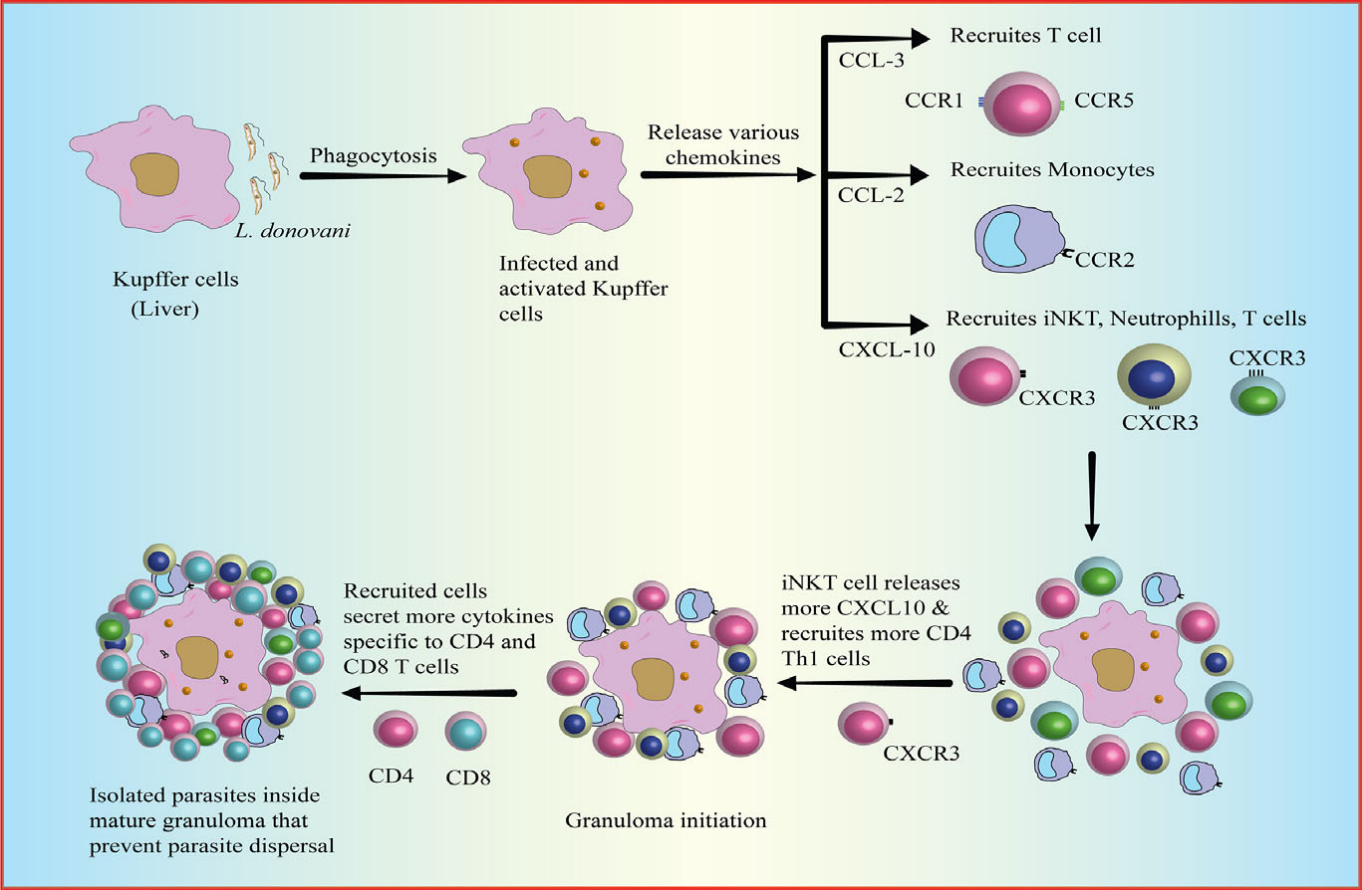
Formation of Granuloma. Granulomas are formed as a response to infection, such as around Kupffer cells in the liver, to elicit a targeted immune response to eliminate parasites and prevent dissemination. Kupffer cells post-infection via phagocytosis (a) get activated and thereafter release chemokines such as CCL2, CCCL3, and CXCL10 that assist in the recruitment of immune cells like monocytes, T cells, neutrophils, and iNKT cells to the site of infection (b) leading to accumulations of the immune cell around the site of infection (c). iNKT cells are essential for the expression of CXCL10, an inflammatory chemokine, which recruits iNKT cells and initiates granuloma formation (d). Similarly, altogether recruited cells secrete chemokines that attract lymphoid cells, contributing to immune defence against *Leishmania* parasites. Hepatic CD4^+^ and CD8^+^ T cells are crucial in the liver immune response against leishmaniasis by the formation of granuloma around the site of infection (e). [CCL3: chemokine ligand 3; CCL2: chemokine ligand 2; CXCL10: C-X-C motif chemokine ligand 10; CCR: beta-chemokine receptors; iNKT: invariant natural killer T cells].

The formation and maturation of granulomas in response to infection, including leishmaniasis, are dependent on active cell recruitment (Ref. 176). Granulomas are complex inflammatory structures that develop around infected cells, such as Kupffer cells in the liver. It includes a variety of immune cells, including several types of T cells, particularly CD4^+^ T cells that produce protective IFN-γ (Ref. 6). Kupffer cells phagocytosed parasites but were unable to eliminate them solely and as the formation of mature granuloma progresses, T cells become a central component of the mature granuloma and contribute to the leishmanicidal activity of infected Kupffer cells (Refs. 177–179). These cells work together to provide a targeted immune response and prevent the parasites from spreading to other tissues. Chemokines play a crucial role in orchestrating the formation and maturation of granulomas. They regulate the recruitment and infiltration of various immune cells into the granuloma, allowing for a more effective immune response against the infection. Chemokines secreted by activated Kupffer cells, such as CCL2, CCL3, and CXCL10 (Ref. 180) attract immune cells like monocytes, T cells, neutrophils, and invariant natural killer T (iNKT) cells to the site of infection. iNKT cells, upon activation, are necessary for the sustained expression of CXCL10, an inflammatory chemokine that binds to CXCR3 and recruits some more iNKT cells. This promotes the initiation of the granuloma formation where iNKT cells are predominantly present (Refs. 181, 182). In an in vivo model of VL, CXCL10 was shown to generate a protective pro-inflammatory environment by upregulating Th1 cytokines (IL-12, IFN-γ, TNF-α) and downregulating anti-inflammatory IL-10 & TGF-β cytokines (Refs. 183, 184), creating an environment favourable for the immune response against the infection. In the inflammatory environment, the presence of IFN-γ cytokine can induce the expression of the inflammatory chemokine CXCL9, CXCL10 and CXCL11 which attracts some more CXCR3^+^ T cells to the site of infection (Ref. 185), suggesting a positive feedback loop around these chemokines and IFN-γ. Other chemokines, such as CCL19, CCL27, CXCL16, CCL9, and CCL25, that selectively attract lymphoid cells have also been observed to be expressed during an early infection (Ref. 181). The recruitment of T cells contributes to the immune defence against parasite *L. donovani* by promoting the maturation of granulomas and facilitating the elimination of infected cells (Ref. 186). The protective inflammatory environment created due to accumulated T cells (CD4^+^ T cells, CD8^+^ T cells etc.) highlights their importance in the liver immune response against parasites as observed in an experimental mice model infected with *L. donovani* (Refs. 187–189) (Figure 3).

Overall, the interplay between the chemokine system and T cells is critical for the development and function of hepatic granulomas in leishmaniasis. Understanding the specific chemokines and receptors involved in T cell recruitment and function within granuloma provides insights into the potential points of intervention that help in pathogen clearance.

## Altered chemokine profiles during Leishmaniasis: protection vs parasite persistence

*Leishmania* infection induces the expression of several chemokines and chemokine receptors that promote the migration of specific immune cell subsets. The parasites have the ability to modify the expression of chemokines and chemokine receptors, either upregulating or downregulating them, in order to persist within the host (Refs. 190, 191). This suggests that the modified chemokine expression profiles and impaired immune cell migration are related to the disease and its pathogenesis. In the liver of *L. donovani-infected* BALB/c mice, the resolution of infection initially occurs independently of T cells. This suggests that mechanisms other than T cell-mediated responses are involved in controlling the infection during the early stages. However, as the infection progresses, T-cell dependence becomes crucial for the expression of chemokines and the recruitment of inflammatory cells (Ref. 192). Immune cells are likely to migrate from secondary lymphoid organs to sites of higher chemokine concentration during an immune response.

The alteration in chemokine receptor expression can modulate the migratory properties of T cells. Activated T cells exhibit a switch in chemokine receptor expression from constitutive to inflammatory, contributing to the altered migration of these cells. Specific chemokines such as CCL2 (MCP-1), CCL3 (MIP-1α), and CCL4 (MIP-1β) are known to stimulate the migration of activated CD4^+^ and CD8^+^ T lymphocytes to the infected sites where an immune response is being mounted (Refs. 193, 194). The parasite *L. major*, which causes CL, has been demonstrated to influence the mRNA expression of chemokines such as CCL2 and CXCL8, providing more evidence that the infection affects chemokine expression (Ref. 195). CCL2 that interacts with CCR2 is found to be upregulated in early lesions of human CL infection with *L. braziliensis* when compared with their healthy controls (Ref. 196). CCL2 is believed to be a biomarker of cure because it was upregulated in cured VL patients (Refs. 197). CCL3 and CCL5 (RANTES), which are ligands for CCR1 and CCR5, selectively attract Th1 cells and are produced in high levels during a Th1 response (Refs. 198, 199). Elevated levels of CCL5 have been reported in the *L. major*-infected mice model and correlated with parasite control (Ref. 200). Although increased CCL3 expression is linked to early control of parasitic load and the establishment of an anti-leishmanial milieu, it also facilitates parasite survival during the later phases of *L. donovani* infection (Ref. 178). CCL7 (MCP-3) interact with several receptors (CCR1, CCR2, CCR3, CCR5 and CCR10) and was found to be upregulated during *L. major* infection (Ref. 201) and promote Th2 cell migration (Ref. 202). Chemokine expression profiles have also been used to define different clinical forms of Leishmaniasis. Elevated levels of chemokines such as CCL2, CXCL9, and CXCL10 have been observed in the lesions of patients with localized CL while diffused CL patients have upregulated CCL3 (Ref. 203). Upregulation of these chemokines may indicate an attempt to recruit immune cells and initiate an effective immune response despite the disease progression.

In human MCL caused by *L. braziliensis*, there is an increase in mRNA and serum levels of CXCL10. This upregulation of CXCL10 suggests its involvement in the immunopathogenesis (Ref. 204). CXCL9 and CXCL10 expression is also upregulated during active VL which is known to recruit CXCR3^+^ Th1 cells and may contribute to tissue damage and disease severity (Refs. 191, 205, 206). The increased expression of CXCL10 during a long infection period in *L. donovani*-infected mice further supports its role in the immune response against the parasite (Ref. 207). Further, the reduced presence of CXCR3^+^ Treg cells in CXCL10^−/−^ *L. donovani*-infected mice suggests that CXCL10 is important for their recruitment. This altered Treg cell trafficking may contribute to a decrease in the regulatory mechanisms that control the immune response against the parasite, ultimately resulting in a lower parasitic load (Ref. 208). This suggests that CXCL10 is involved in creating a favourable immune environment for parasite control.

While information on T cell trafficking during *Leishmania* infection may be limited, the role of certain chemokine receptors expressed on T cells has been investigated in the context of Leishmaniasis. Some important chemokine receptors and their potential roles in different phenotypes of Leishmaniasis are discussed below:

### 1. CXCR3

In *L. infantum* infected mice, the Cxcr3 gene is found to be associated with the activated T lymphocytes, including effector cells and regulatory cells, suggesting their initial migration towards the affected spleen (Ref. 178). It is a crucial chemokine receptor involved in the trafficking of activated CD4^+^ T cells and CD8^+^ T cells during infection (Refs. 209, 210). It interacts with its ligands, CXCL9 (MIG), CXCL10 (IP-10), and CXCL11 (I-TAC), and promotes integrin activation and immune cell migration (Refs. 211). CXCR3 is a remarkable marker of Th1 cells and their lower expression causes less trafficking of Th1 cells to the inflamed tissues during VL. It leads to less IFN-γ production that affects the host’s protective response against the parasite (Ref. 212). In an experimental model of VL, a reduced number of CXCR3^+^ CD4^+^ T cells have been observed in the spleen compared to the liver during the chronic phase of infection, and this impairment is associated with a high parasitic burden in the organ, suggesting the importance of CXCR3 in host immunity. However, their upregulated expression on T cells does not prevent from developing VL as studied in transgenic mice that overexpressed CXCR3 on all T cells (Ref. 213). A prior study on CXCR3^−/−^ C57BL/6 mice has shown that CXCR3 plays a crucial role in resolving disease during *L. major* infection as it is necessary for T cell trafficking in the skin, but it is not essential during *L. donovani* infection, as mutant mice are still able to recruit T cells to the affected organs at later stages and exhibit a Th1 response, to effectively clear the infection similar to CXCR3^+/+^ mice (Ref. 214). This suggests that the CXCR3 is necessary for T cells trafficking in the skin during *L. major* infection. Also, a higher frequency of infiltrating cells was IFN-γ-producing Th1 and Tc1 cells expressing CXCR3, accounting for the resolution of dermal lesions (Ref. 215).

### 2. CCR1

CCR1 belongs to the beta-chemokine receptor family which interacts with several ligands, including Regulated on Activation Normal T Expressed and Secreted Protein (RANTES/CCL5), Macrophage Inflammatory Protein 1 alpha (MIP-1α/CCL3), Monocyte Chemoattractant Protein 3 (MCP-3/CCL7), and Myeloid Progenitor Inhibitory Factor-1 (MPIF-1/CCL23). While CCR1 expression is preferentially found on CD4^+^ Th1 cells (Ref. 216) and is involved in recruiting effector cells to infection sites, the specific role of CCR1 in the immune response to Leishmaniasis can vary depending on the context and the specific species of *Leishmania* involved. In C57BL/6 mice infected with *L. major*, it was found that CCR1 could actually contribute to susceptibility to CL, associated with an enhanced production of interleukin-4 (IL-4) and interleukin-10 (IL-10) which suggests a shift towards a Th2 immune response (Ref. 217). Previous research has revealed the expression of CCR1 by CD8^+^ T cells (Ref. 218) in different diseases but no studies have been conducted to investigate this expression in the context of Leishmaniasis.

### 3. CCR2

CCR2 is the main receptor for the chemokine monocyte chemoattractant protein 1 (MCP-1), also referred to as CCL2. It also binds with other chemokines such as CCL7 and CCL12. When CCR2 interacts with its ligands, it initiates signalling pathways that increase intracellular calcium levels (Ca^2+^) and lead to the recruitment of memory T cells, monocytes, and dendritic cells to inflamed tissues (Refs. 219–221). CCR2 has been shown to promote the differentiation of T cells into Th17 cells, which are characterized by the production of interleukin-17 (IL-17) and contribute to inflammatory responses in the colon. While in the absence of CCR2 signalling as studied on RAG1^−/−^ immunocompromised mice transferred with CCR2^−/−^ T cells, there is an increase in the conversion of T cells into FoxP3^+^ regulatory T cells (Tregs), which are involved in immune tolerance and suppression of immune responses (Ref. 222). It suggests that the presence and absence of CCR2 signalling play an important role in the differentiation of T cells.

The association between CCR2 and T cells in the context of Leishmaniasis has not been extensively studied compared to other chemokine receptors such as CCR1, CCR3, and CXCR3. The research focus has primarily been on these other receptors and their involvement in the immune response to *Leishmania* infection. However, considering the role of CCR2 in recruiting monocytes and dendritic cells, it is plausible that CCR2 may also play a role in modulating T-cell responses during *Leishmania* infection. The recruitment and activation of these antigen-presenting cells by CCR2 may influence the subsequent T-cell responses and the overall immune response against the parasite. To fully understand the specific involvement of CCR2 in T cell responses and its impact on the immune response to Leishmaniasis, further studies are needed.

### 4. CCR4

CCR4 is primarily expressed in activated T cells, particularly Th2 cells, antigen-specific skin-homing T cells and Treg cells (Refs. 223, 224). When CCR4 interacts with its ligand, CCL17 (also known as thymus and activation-regulated chemokine; TARC), it can lead to an increase in intracellular calcium levels (Ref. 225). While CCR4 is predominantly expressed in Th2 cells, other cell types, which may not necessarily be IL-4 producers, can also express CCR4. In human VL, higher expression of CCR4 on regulatory T cells (Tregs) has been observed, and this increased expression may contribute to the accumulation of Tregs in the bone marrow of VL patients. The accumulation of CCR4-expressing Tregs in the bone marrow may suppress local effector T cell responses, thereby dampening the immune response against *Leishmania* parasites in this compartment (Ref. 129). In late localized CL caused by *L. braziliensis and L. amazonensis*, it has been reported that there is an increase in CCR4 expression on Tregs that facilitate their recruitment and accumulation in the affected skin tissue. This accumulation of CCR4-expressing Tregs suggests a potential role for CCR4 in regulating immune responses and contributing to the immunosuppressive environment at the inflammatory sites (Refs. 196, 226). These cells produce significant amounts of IL-10 and TGF-β, which regulate the functions of effector T cells and thus the disease outcome (Ref. 227). CCR4-expressing Th2 cells and Treg cells promote the development of PKDL (Ref. 119). The trafficking of CCR4 expressing CD8^+^ T cells in response to CCL17 and CCL22 in the dermal lesion has been reported during PKDL (Ref. 160).

### 5. CCR5

CCR5 is a chemokine receptor that specifically binds to chemokines such as regulated on activation, normal T cell expressed and secreted (RANTES), macrophage inflammatory protein 1 alpha (MIP-1α), and macrophage inflammatory protein 1 beta (MIP-1β). Its expression on cells is indicative of their activation state, and it is known to be expressed at higher levels on Th1 cells (Ref. 228) which can be upregulated by the cytokine interleukin-2 (IL-2) (Ref. 229). In early infection with *L. donovani*, mice lacking CCR5 (CCR5^−/−^; hybrid mice) showed impaired interferon-gamma (IFN-γ) responses following T cell receptor (TCR) stimulation (Ref. 230). This suggests that CCR5 plays a role in facilitating IFN-γ production by T cells during the early stages of *Leishmania* infection and participates in the host defence mechanism. CCR5 has also been identified as a crucial marker for the migration of naturally occurring regulatory T cells (Tregs) to infected dermal skin during chronic cutaneous infection caused by *L. major* parasite (Refs. 95, 231). This indicates that CCR5 is involved in the recruitment of Tregs to sites of infection, potentially influencing immune regulation and the balance between effector and regulatory responses and promoting parasite persistence.

Furthermore, in other protozoan infections like Chagas disease caused by *Trypanosoma cruzi*, CCR5 expression has been found to be upregulated on CD4^+^ and CD8^+^ T cells. This upregulation of CCR5 is associated with increased trafficking of these T cells to pathological sites and has been correlated with pathogenic conditions (Ref. 232). Overall, CCR5 plays a role in immune responses by regulating T cell activation, migration, and cytokine production in various infectious diseases, including Leishmaniasis and Chagas disease. Its involvement in these processes highlights its significance in modulating immune cell responses and potentially impacting disease outcomes.

### 6. CCR6

CCR6 is a chemokine receptor that regulates the migration of T cells during homeostatic and inflammatory responses (Ref. 233). Interaction between CCR6 and ligand CCL20 leads to an increase in intracellular calcium ion levels, which then triggers intracellular signalling and cellular responses (Ref. 234). CCR6 is expressed on both anti-inflammatory regulatory T cells (Tregs) and pro-inflammatory Th17 cells during inflammatory diseases, and promotes immune regulation or inflammatory responses, respectively (Refs. 235–237). It plays a role in the recruitment and migration of T cells to specific sites of inflammation (Ref. 238). In the context of *L. major* infection, studies using CCR6-deficient (CCR6^−/−^) mice have shown that CCR6 is involved in the trafficking of Treg cells. CCR6 deficiency resulted in hampered migration of Treg cells and an increase in inflammatory responses while having no effect on Th17 cell migration (Ref. 239). This indicates that CCR6 is important for the proper trafficking and localization of Treg cells to the site of infection to prevent disease severity during *L. major* infection. However, further research is needed to fully understand the precise mechanisms by which CCR6 influences T-cell migration and the implications for the immune response to *Leishmania* and other inflammatory conditions.

### 7. CCR7

CCR7 is a crucial receptor involved in the homing of cells to lymph nodes and interacts with its ligands, CCL19 and CCL21. CCR7 plays a significant role in regulating the migration and homeostasis of memory T cells in lymphoid tissues where priming of antigen-specific T cells occurs (Refs. 240, 241). During VL, an increase in CCR7 expression has been reported in peripheral blood mononuclear cells (PBMCs). As CCR7 is a marker of naïve and central memory T cells (Tcm), the upregulated CCR7 may contribute to their trafficking in lymphoid tissues where naïve cells encounter antigen-presenting cells (APCs) during the course of the infection and Tcm cells reside within SLOs and rapidly respond upon re-exposure to antigen (Ref. 212). Reduced expression of CCR7 on activated dendritic cells (DCs) reduces their migration to the draining lymph node and is found to promote pathogenesis during CL and VL (Refs. 242, 243). In cured CL patients, it has been observed that CCR7^−^ CD4^+^ effector memory T (Tem) cells are present in larger numbers. These cells are capable of producing interferon-gamma (IFN-γ) when stimulated with soluble *Leishmania* antigens (SLA). The presence of CCR7^−^ CD4^+^ Tem cells producing IFN-γ suggests a potential role for these cells in the immune response and resolution of CL (Ref. 244). These studies highlight the dynamic regulation of CCR7 and its potential implications in the immune response against *Leishmania* parasites.

## Factors shaping chemokines and chemokine receptors’ expression during Leishmaniasis

The dysregulation of the chemokine system during infection may result from a complex interplay between the parasite, host immune cells, and the local microenvironment. Interaction between the host and the *Leishmania* parasite can lead to the modulation of the chemokine system. *Leishmania* has been reported to secrete molecules that can degrade chemokines, such as CXCL1, resulting in the downregulation of their expression (Ref. 245).

However, various factors such as cytokine levels, epigenetic changes, and mutations contribute to the modulation of chemokine receptor expression and downstream signalling pathways. These factors can directly or indirectly influence the behaviour of the chemokine profile during infection. The possible causes for the altered chemokines profile during *Leishmania* infection have been discussed below:

### 1. Cytokines

There is a complex interplay between cytokines and the expression of chemokines and chemokine receptors, which contributes to the heterogeneity observed in the immune response during Leishmaniasis. Cytokines such as IFN-γ, IL-10, TGF-β, TNF-α, and IL-17, among others, play a crucial role in regulating the expression of chemokines and chemokine receptors on immune cells, ultimately shaping the cellular landscape at the site of infection (Table 4). IFN-γ, for example, has been shown to induce the expression of chemokines such as CXCL9, CXCL10, and CXCL11 (Ref. 260). Therefore, changes in the expression levels of CXCL9 & CXCL10 observed during Leishmaniasis (Refs. 212, 261) maybe due to the influence of IFN-γ. Additionally, cytokines like IL-2, IL-4, IL-7, and IL-15, which utilize the common gamma c (γc) chain receptors, can induce CXCR4 expression on T cells through the JAK/STAT signalling pathway (Ref. 262). The role of IL-4 in modulating chemokine expression has also been demonstrated. Blocking IL-4 in *L. major*-infected dermal tissue resulted in increased expression of Th1 cell-recruiting chemokines such as CXCL9, CXCL10, CXCL11, and CCL5, coinciding with increased IFN-γ production at the inflamed region (Ref. 263).

**Table 4.** Influence of cytokines on chemokines/and receptors, T cell profiles, and outcome of infection during Leishmaniasis.

S.No.	Cytokines	Affect chemokines/and chemokine receptor expression	Impact on specific T-cell subset	Outcome of *Leishmania* infection	References
**1**	IFN-γ	CXCL9, CXCL10, CXCL11 **(↑)**	more CXCR3^+^ Th1 cells trafficking	resolution of infection	(246; 247; 248; 249; 250)
**2**	IL–2, IL–7, IL–15	CXCR4 **(↑)**	express on central memory T cells (CD4^+^ T cell subset); induces T cell chemotaxis	parasite may facilitate HIV infection of CD4^+^ T cells during *Leishmania*-HIV coinfection	(251; 252; 253)
**3**	IL–4	CXCR4 **(↑);** CXCL9, CXCL10, CXCL11, CCL2, CCL5, CCR5 **(↓)**	less trafficking of Th1 cells	less IFN-γ production in *L. major* infected dermal tissue; shows pathogenic T cell response	(251; 252; 254; 255)
**4**	IL–17	C-X-CL types	recruit more Th1 cells	protective role in VL patients; skin inflammation in CL	(125; 256; 257)
**5**	IL–10	CCL5, CCL2 **(↓)**	less Th1 cell migration	reduces Th1 cell development and effector functions; promote parasite persistence and pathogenesis	(258; 259)

Furthermore, TGF-β, which is increased during *Leishmania* infection, can inhibit macrophage activation and contribute to increased susceptibility to the disease (Ref. 264). TGF-β has also been shown to inhibit CCR3 expression, which is associated with decreased Th2 cell development. Conversely, IFN-α, a type I interferon, decreases CCR3 and CCR4 expression while increasing CXCR3 and CCR1 expression, promoting Th1 cell polarization by upregulating these chemokine receptors (Ref. 185). IL-17, a proinflammatory cytokine, can induce the production of CXCL chemokines, which recruit neutrophils and Th1 cells to the site of infection, thus showing its protective role in patients with VL (Ref. 132). A positive correlation was found between IL-17/CCL3 and IL-17/CCL4 in patients infected with *L. guyanensis* (Ref. 144). On the other hand, IL-10, which is responsible for impairing inflammatory immune responses, has been shown to decrease the production of chemokines such as CCL5 and CCL2 in *L. amazonensis*-infected mice (Ref. 265).

Therefore, the presence of various cytokines in the microenvironment at the site of infection, directly and indirectly, influences the outcome of the disease by regulating the expression of chemokines and chemokine receptors, ultimately shaping the immune response and cellular profiles observed in Leishmaniasis.

### 2. Epigenetics

The expression of chemokines and chemokine receptors can be modulated by the parasite through various mechanisms, including the alteration of host gene expression and epigenetic pathways (Ref. 238). Endogenous processes such as DNA methylation and histone modification can inhibit the expression of chemokines and chemokine receptors, resulting in decreased infiltration of immune cells (Refs. 266–268). *Leishmania* has been shown to produce effector molecules such as exosomes or microRNA that can modify the host immune transcriptome and induce changes in chemokine expression (Refs. 269, 270). Additionally, the parasite has been shown to regulate chemokine expression through the modulation of host microRNA levels. Several chemokines, including CCL2, CCL5, and CXCL10 found to be inhibited by the activity of upregulated miRNA in *L. major* infected macrophages (Ref. 271). These epigenetic mechanisms could contribute to the fluctuations observed in the expression levels of the chemokines profile at different stages of infection. It is likely that *Leishmania* employs these mechanisms to evade the host immune system and establish persistence within the host. However, the role of epigenetic regulation in parasitic diseases, including Leishmaniasis, is not yet extensively studied. Similar mechanisms have been observed in certain cancers, such as pancreatic cancer, where abnormal methylation can lead to lower expression of CXCR4 (Ref. 272).

Further research into the epigenetic modulation of the chemokine system during Leishmaniasis and other parasitic diseases is necessary to better understand the mechanisms employed by the parasite to manipulate the host immune response as an evasion strategy, or by the host that employs epigenetic mechanisms as a protective response against parasitic disease.

### 3. Mutation

The N-terminal region of chemokines is crucial for their biological activities and interaction with chemokine receptors (Ref. 273), mutations in this region can disrupt their binding to their respective receptors, rendering them unable to activate the receptors. For instance, mutation at a phosphorylation site can reduce receptor phosphorylation, impair β-arrestin binding, and subsequently reduce receptor internalization in response to ligand binding (Ref. 274). Mutation in residues of two CKRs failed to oligomerize together and cells expressing such receptors do not migrate even in the presence of their cognate antigens as observed in the case of CCR7 and CCR5 (Refs. 117, 275).

Mutation at the gene level is also capable of making changes in chemokines/and chemokine receptors expression, potentially resulting in their aberrant expression. It has been reported that *Trypanosoma cruzi-infected* patients with no cardiac disease showed lower CCR5 expression than those with cardiac disease due to a higher frequency of point mutations found in the promoter region (Ref. 276). As it is known that CCR5 expression is associated with protective Th1 cells, an increased frequency of mutation in CCR5/Δ32 alleles has been reported in the lesions of American CL (ACL) patients which suggests that this mutation may reduce Th1 cells trafficking to the lesions and contribute to the pathogenesis in ACL patients (Ref. 277).

While mutations have not been extensively studied in the context of Leishmaniasis, they have the potential to play a role in modulating the immune response. Further research is needed to elucidate the specific roles of mutations in the context of Leishmaniasis and their impact on the chemokines profile and immune response.

## Modulation of chemokine machinery: plausible mechanisms

In addition to the factors that have been discussed above, there are some other mechanisms that influence the expression of chemokine machinery which include chemokine availability, receptor desensitization, decoy receptors, allosteric effects, post-translational modification and so forth. However, these aspects have not been investigated in the context of Leishmaniasis, and they may be plausible mechanism of aberrant expression observed in the chemokine profiles which should be further investigated. The most significant mechanisms which have not been explored yet are discussed below:

### 1. Chemokine availability and desensitization

The process of desensitization is an important mechanism for regulating chemokine receptors (CKRs). Phosphorylation of CKRs triggers a series of events that regulate their signalling and trafficking. Upon phosphorylation, CKRs become uncoupled from G proteins and recruit β-arrestin. β-arrestin binding blocks further coupling to G proteins and facilitates the internalization of the receptor via clathrin-coated pits (Ref. 278). This internalization process is important to prevent chemokine overstimulation and allows for directional cell migration. Homologous desensitization, which is chemokine-dependent, involves the internalization and degradation or redistribution of the receptor. It plays a crucial role in regulating the chemokine receptor response and maintaining appropriate chemotactic responses (Ref. 279). Heterologous desensitization, on the other hand, is chemokine-independent and leads to the uncoupling of G-protein and downregulation of chemokine receptors. It is usually due to cross-talk between two CKRs, where signalling of one CKR on chemokine binding impacts another chemokine-free CKR and modulates their chemotactic response towards chemoattractant by downregulating them (Ref. 280). It was shown that CCL2 caused a reduction in the expression of CCR2 on the surface of monocytes over time, due to the desensitization mechanism (Ref. 281). Another study on human cells revealed the existence of a desensitization mechanism where CCL22 binding leads to the internalization of CCR4 and hence reduces surface expression on Th2 cells (Ref. 282). However, no studies have been performed in case of leishmaniasis. The reduction in chemokine receptor expression and the lower number of T cells recruited to the infected tissue during Leishmaniasis may be attributed to these desensitization phenomena. The expression of chemokines and chemokine receptors are interdependent. It has been reported previously that high chemokine levels lead to lower CKR expression specific to that chemokine (Ref. 191). Particularly for chemokines that signal through multiple receptors, the absence of one receptor can result in high levels of circulating chemokines, which may reduce the availability of alternate receptors due to ligand-mediated desensitization (Ref. 283). These processes highlight the dynamic interplay between chemokines and their receptors, and the regulation of chemokine receptor expression and responsiveness is critical for appropriate immune cell recruitment and migration during other inflammatory responses.

### 2. Chemokine scavenging decoy receptors

The presence of non-signalling or silent chemokine receptors acting as ‘decoys and scavengers’ plays an important role in suppressing host inflammatory responses and immunity. The silent receptors compete with the signalling chemokine receptors by binding their ligands with high affinity and thus preventing the cell from activation (Ref. 284). Functional decoy receptors have been reported for inflammatory chemokine receptors such as CCR1, CCR2 and CCR5, in monocytes and dendritic cells and despite increased expression of these chemokine receptors, they do not respond to their ligands (Refs. 285, 286). It has been reported previously that IL-10 may generate chemokine decoy receptors in monocytes and dendritic cells in an inflammatory environment, leading to the termination of the early inflammatory phase in the brain of *L. donovani* infected mice (Ref. 287). Despite little knowledge about decoy receptors in the context of Leishmaniasis and other parasitic disease, investigating their role will contribute to our understanding of infection and the progression of the disease.

The higher expression of chemokine receptors observed during Leishmaniasis may be a host strategy to address the urgent requirement for receptor-based signalling and prevent disease progression. However, the presence of related decoy receptors limits the responsiveness of immune cells to these chemokines. Consequently, despite the higher expression of chemokine receptors, migration to the inflamed zone may be limited. Decoy receptors also act as “scavengers” for chemokines, reducing their availability through intracellular degradation. This mechanism helps regulate proinflammatory chemokines and chemokine receptors. The presence of decoy and scavenger receptors highlights the complexity of the chemokine system and its regulation during infection. Understanding the interplay between signalling and decoy receptors is crucial for deciphering the immune response dynamics.

## Future prospects and concluding remarks

The chemokines and chemokine receptors play a crucial role in immune cell trafficking and the inflammatory responses associated with *Leishmania* infection. Dysregulation of the chemokine system is observed during Leishmaniasis, and investigating the involvement of chemokines and their receptors in disease symptoms helps us understand how effective immune responses are orchestrated and how pathological inflammation develops. The redundancy and large production of multiple chemokines during infection may contribute to the effectiveness of the immune response. Alterations in the expression levels of chemokines and chemokine receptors can potentially serve as diagnostic markers and immunotherapeutic targets. Blocking chemokines and their receptors, particularly the CXC- and CC-chemokines, could be an attractive strategy for immunotherapy, especially during the chronic phase of infection. While the role of the chemokine system in other immune cells in Leishmaniasis has been extensively studied, further exploration of its involvement in T cell trafficking is needed. Additionally, the understanding of the factors responsible for the altered profile of chemokines and chemokine receptors in leishmaniasis is still limited and requires investigation.

Future research should focus on identifying the factors, both derived from *Leishmania* and the host, that contribute to the changes observed in the chemokines and chemokine receptors expression. The properties of the recruited immune cells will ultimately determine the pathogenic condition of the host, making it important to elucidate the underlying mechanisms. In the recent past, targeting chemokines and chemokine signalling pathways using agonistic or antagonistic monoclonal antibodies has emerged as an effective and promising therapeutic approach in cancer patients. This targeted approach, either alone or in combination with conventional drug therapy has shown promising results in modulating the immune response and enhancing anti-tumor immunity. Therefore, targeting the chemokine system as an immunotherapeutic approach also holds promise for the treatment of leishmaniasis. However, further studies, including those specifically investigating T cell chemokine machinery and its role in PKDL, are warranted to advance our understanding and develop effective interventions for this neglected tropical disease.
